# Improving Parameter-Efficient Medical Image Classification with Lesion-Aware Hierarchical Knowledge Distillation

**DOI:** 10.3390/jimaging12090437

**Published:** 2026-09-11

**Authors:** Yarong Liu, Runmei Xie, Xiaolan Xie, Huilin Zheng

**Affiliations:** 1 School of Computer Science and Engineering, Guilin University of Technology, Guilin 541006, China; hblyr@glut.edu.cn (Y.L.); runmeixie@glut.edu.cn (R.X.);; 2 Guangxi Key Laboratory of Embedded Technology and Intelligent System, Guilin University of Technology, Guilin 541006, China

**Keywords:** medical image classification, knowledge distillation, lightweight model, CNN-Transformer, lesion-aware

## Abstract

Compact deep models are attractive for medical image classification, but conventional knowledge distillation mainly transfers class-level predictions and may not adequately preserve lesion-relevant spatial cues. To address this limitation, we propose LaHKD, a lesion-aware hierarchical knowledge distillation framework for parameter-efficient medical image classification. LaHKD enables a lightweight student to learn hierarchical semantic representations together with lesion-focused guidance from a stronger teacher. We evaluate LaHKD on HAM10000 dermoscopic lesion classification and a brain tumor MRI classification benchmark. Across both datasets, LaHKD improves compact-student classification performance, with the clearest lesion-focused spatial benefits observed on HAM10000, where lesion morphology is central to diagnosis and direct lesion supervision is available. On the magnetic resonance imaging (MRI) benchmark, localization analysis is limited to an auxiliary recovered-mask subset and is therefore interpreted as exploratory; under this setting, consistent localization gains are not observed. Overall, LaHKD provides an effective framework for compact medical image classification, with spatial benefits most clearly supported in tasks with reliable lesion supervision.

## 1. Introduction

Medical image classification is a core task in computer-aided diagnosis. Convolutional neural networks (CNNs) excel at local feature extraction [1,2,3] but struggle to capture long-range dependencies. Vision Transformers (ViTs) can model global relationships [4] but rely on large-scale data and have high computational complexity [5,6]. CNN-Transformer hybrid architectures have become an important direction for lightweight medical image analysis [7,8]. However, clinical diagnosis heavily depends on the morphology of local lesions. How to equip a lightweight deployment model with the ability to focus on lesions, just as an expert would, remains a key bottleneck for clinical application.

Knowledge distillation (KD) improves lightweight model performance [9]. Existing techniques have evolved from output-level matching [9] to feature alignment [10,11] and attention transfer [12,13]; cross-architecture distillation has also attracted attention [14,15,16]. HDKD [16] addresses feature mismatch in heterogeneous teacher–student pairs by sharing convolutional stages, but it still does not guide the student to prioritize lesion regions. Recent studies have increasingly recognized that medical image classification benefits not only from global category supervision but also from disease- or region-specific knowledge transfer. For example, lesion-aware distillation has been explored for diabetic retinopathy lesion segmentation, where lesion-specific cues are transferred from a teacher to a compact student model [17]. More recent medical KD frameworks further incorporate clinical priors, privileged information, or region-aware structural knowledge to improve interpretability and reliability, such as clinical-priors-inspired privileged KD for pancreatic lesion classification [18], dynamic weighted KD for skin lesion diagnosis [19], and region-aware cross-modal topological KD for interpretable tumor classification [20]. We therefore position HDKD as a recent medical KD method rather than a conventional distillation baseline.

**Figure 1:**
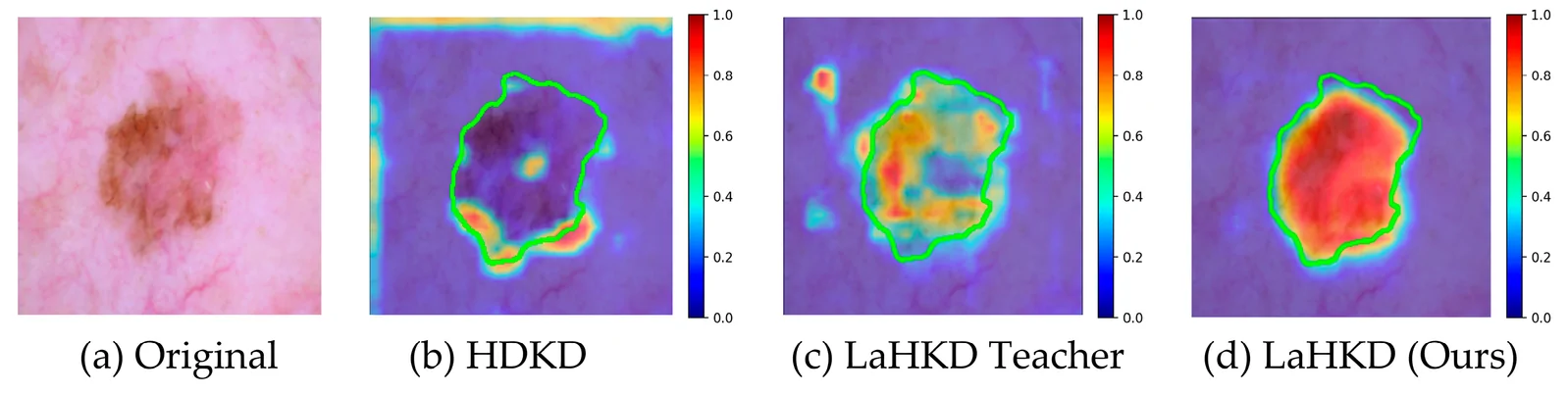
Motivation diagram of LaHKD. (**a**) Original dermoscopic image; (**b**) attention visualization of the reproduced HDKD; (**c**) attention visualization of the LaHKD teacher; (**d**) attention visualization of the proposed LaHKD (ours). All visualizations are min–max normalized independently and use the jet colormap, where warmer colors indicate higher activation.

Nevertheless, most classification-oriented KD methods still transfer knowledge mainly at the logit or global feature level, while explicit lesion-region alignment and lesion-token-level semantic transfer remain insufficiently studied. Specifically, the current medical image KD methods suffer from three limitations: (1) A lack of lesion-targeted distillation objectives: background interference causes the model to overlook small lesions. (2) A lack of lesion-aware optimization in lightweight Transformer heads: uniform allocation of computational resources dilutes diagnostic semantics. (3) A failure to jointly model lesion spatial importance and hierarchical semantic differences: static equally weighted losses are used. As shown in Figure 1, the reproduced HDKD leads to diffuse attention, whereas introducing lesion spatial priors enables more lesion-focused attention. Each heatmap is min–max normalized independently and visualized using the jet colormap (0.0–1.0, as shown by the colorbars), where warmer colors indicate higher activation. Because each heatmap is normalized independently, color intensity should be evaluated within each individual image to show relative spatial distributions, rather than directly comparing absolute values across different subplots.

To address these challenges, we propose LaHKD, a lesion-aware hierarchical knowledge distillation framework that incorporates lesion spatial priors at three systematic levels: feature encoding, architectural attention, and multi-stage knowledge transfer. The framework consists of: (1) the lesion-aware feature enhancement and compression module (LaFEC) integrates data-driven channel attention with lesion-guided spatial modulation to enhance classification-relevant representations; (2) the lesion-aware lightweight Transformer head (LaLT) replaces standard self-attention with a smooth lesion bias and introduces a learnable lesion token to improve global modeling efficiency; (3) the lesion-aware hierarchical multi-stage distillation strategy (LaHMD) aligns the teacher and student at the logit, feature, and semantic levels, enabling more faithful transfer of lesion-related knowledge.

This work is therefore motivated by a central research question: can a compact medical image classifier approach teacher-level accuracy while explicitly learning lesion-aware spatial and semantic representations through hierarchical knowledge distillation? Our main contributions are as follows:

1. We propose LaHKD, a lesion-aware hierarchical knowledge distillation framework for parameter-efficient medical image classification, designed for heterogeneous CNN teacher and CNN-Transformer student architectures to reduce background interference in compact models.
2. We design two lesion-aware architectural modules, LaFEC and LaLT, which enhance lesion-relevant local representation learning and efficient global context modeling, respectively.
3. We introduce a hierarchical multi-stage distillation strategy, which transfers output-level, feature-level, and lesion-token-level knowledge from the teacher to the student.
4. We conduct comprehensive experiments and ablation studies on HAM10000 and a brain tumor MRI benchmark, showing that LaHKD consistently improves compact classification performance, while lesion-focused spatial benefits are most clearly supported in the dermoscopic setting with direct lesion supervision.

From the perspective of imaging science, LaHKD provides an alternative lesion-aware training workflow in which lesion representations are optimized jointly with classification, thereby reducing reliance on purely post hoc visualization.

## 2. Related Work

### 2.1. Lightweight Networks and Medical Image Analysis

CNNs have long dominated medical image classification due to their local receptive fields and parameter sharing. Lightweight models such as MobileNet [21], ShuffleNet [22], and EfficientNet [3] have been widely applied [23], but they cannot model long-range dependencies. ViT [4] compensates for this via self-attention; DeiT [5] introduces a distillation token; Swin Transformer [6] uses shifted windows. Hybrid architectures like CoAtNet [7], MobileViT [8], and PVT [24,25] have shown advantages in medical images [26], but their Transformer components lack specialized attention mechanisms for sparse lesion distributions.

### 2.2. Evolution of Knowledge Distillation

Hinton et al. proposed KD [9], followed by FitNets [10], attention transfer [11], relational distillation [12], and multi-stage methods [27,28]. In cross-architecture distillation, DeiT [5], DearKD [13], and CSKD [14] attempt to transfer CNN knowledge to ViTs, but feature space differences are large. HDKD [16] addresses this by sharing convolutional stages, yet it still does not guide the student to prioritize the lesions. Medical KD methods [29,30] mostly follow generic designs from natural images and lack explicit lesion modeling.

The recent literature has substantially expanded this direction. KDLight [31] develops a lightweight KD framework for medical image classification, and a multi-teacher KD with ensemble strategy has been proposed for efficient brain tumor classification in resource-constrained environments with explainable AI [32]. In dermoscopy, MANO-UniDerm [33] combines multi-scale attention with KD in a lightweight multimodal skin-lesion framework; DERMAE [34] leverages conditioned latent diffusion and MAE-based distillation to alleviate class imbalance while transferring representations to a mobile-friendly ViT; dynamic weighted KD [19] learns sample-wise weighting based on the teacher’s prediction entropy for skin lesion diagnosis; multi-stage KD with layer fusion has also been applied to skin cancer classification [35]; and Agentic MobileViT [36] integrates dynamic KD with replay regularization for multi-dataset skin lesion classification. These methods demonstrate the growing interest in medical KD, but they mostly transfer knowledge at the logit, sample-weighting, or global feature level, and none explicitly injects lesion priors into the distillation objective or aligns lesion-token-level semantics between teacher and student.

### 2.3. Lesion-Aware and Region-Aware Methods

LACNN [37] employs attention mechanisms to direct model focus toward lesion regions, yet requires pixel-level annotations during training. Attention transfer [11] and HDKD [16] eliminate annotation requirements; however, their spatial priors are derived purely from data statistics and lack explicit lesion targeting. Sevinc [38] and Manivannan [39] demonstrated the efficacy of distillation for medical image tasks but did not incorporate lesion-aware supervision. Ngan [40] introduced a neural-symbolic distillation framework, though it depends on manually defined rules. Lesion- or region-aware knowledge transfer has been explored in several recent settings. LKD [17] transfers lesion-specific cues for diabetic retinopathy lesion segmentation, demonstrating the value of lesion knowledge, albeit in a segmentation setting with pixel-level supervision. CPKD [18] introduces clinical-priors-inspired privileged knowledge distillation for reliable pancreatic lesion classification, relying on a privileged modality as auxiliary supervision. REACT-KD [20] performs region-aware cross-modal topological distillation for interpretable tumor classification, transferring supervision from high-fidelity multi-modal sources into a lightweight CT-based student. In dermoscopy, MTA-Net [41] employs multi-scale triplet attention for skin lesion classification, and Thoraxformer [42] embeds lesion awareness in a hybrid Transformer for multi-label chest X-ray classification without dense annotations. Compared with these methods, LaHKD operates in a classification-only setting without privileged modalities or pixel-level annotations at inference and is distinguished by aligning lesion tokens between teacher and student as an explicit third distillation level.

## 3. Methodology

**Figure 2:**
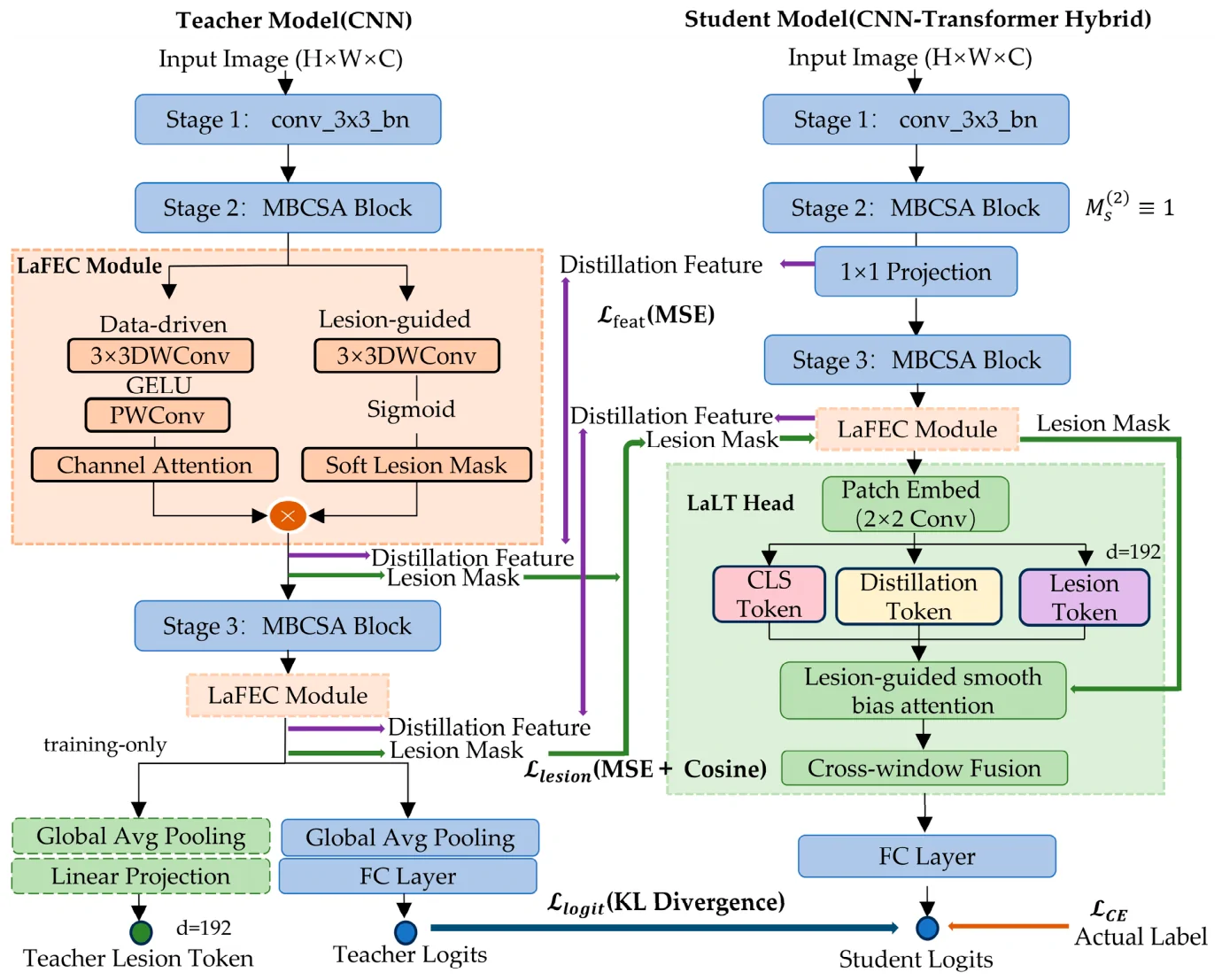
Overview of the LaHKD framework. The teacher-side lesion token is constructed from the Stage-3 LaFEC feature using lesion modulation, global average pooling, and a linear projection. This branch is used only during distillation. Purple arrows denote feature-level distillation; green arrows denote learned lesion-aware attention maps (including the student-side map injected into the LaLT head); blue arrows denote logit distillation; and orange arrows denote supervision from ground-truth labels.

LaHKD follows a heterogeneous knowledge distillation framework, where a pure CNN teacher transfers lesion-aware knowledge to a compact CNN-Transformer hybrid student. As shown in Figure 2, the teacher is first trained with cross-entropy loss and then frozen. During student training, the student is optimized by combining classification supervision, output-level distillation, lesion-aware feature distillation, and lesion-token alignment. During inference, only the student network is retained, and all teacher-side branches are removed.

### 3.1. LaFEC: Lesion-Aware Feature Enhancement and Compression

LaFEC is designed to enhance lesion-relevant representations while compressing intermediate feature maps for efficient downstream modeling. Given an intermediate feature map, LaFEC predicts a lesion-aware spatial attention map and uses it to modulate the feature response. The teacher applies LaFEC at both Stage 2 and Stage 3. In contrast, the lightweight student applies LaFEC only at Stage 3, while Stage 2 uses a simple 1 × 1 projection for channel alignment. This design avoids introducing an unstable learned lesion-aware attention map at low-level student features and reduces computational cost.

Background tissues and artifacts constitute redundant information. Existing attention mechanisms are data-driven and task-agnostic, and are prone to misallocating high responses to normal structures. LaFEC introduces an end-to-end self-learned lesion spatial prior to modulate feature responses, achieving background suppression and lesion enhancement.

LaFEC consists of two parallel branches: a data-driven branch and a lesion-guided branch. The lesion-guided branch takes the input feature map $X\in R^{B\times C\times H\times W}$ and applies a 3 × 3 depthwise separable convolution to compress it to a single channel, followed by a sigmoid activation to obtain a learned lesion-aware attention map:

(1) $$\begin{array}{c} A_{lesion}=\sigma (Conv_{3\times 3}^{DW,C\to 1}(X))\in (0,1{)}^{B\times 1\times H\times W} \end{array}.$$

This mask is learned entirely from end-to-end gradients of the classification task and requires no pixel-level segmentation annotations. The data-driven branch computes channel attention:

(2) $$\begin{array}{c} A_{data}=\sigma (W_{2}\cdot \phi (W_{1}\cdot \phi (Conv_{3\times 3}^{DW}(X))))\in (0,1{)}^{B\times C\times H\times W} \end{array},$$

where ϕ(·) is the GELU activation function, and $W_{1}$ and $W_{2}$ are pointwise convolution weights for channel compression and restoration, respectively.

Throughout the paper, ground-truth lesion masks refer to the official segmentation annotations, which are used only for evaluation and are never used to train LaHKD. Learned lesion-aware attention maps denote the spatial priors produced internally by LaFEC from end-to-end classification supervision. Lesion priors refer, in this work, to this learned lesion-focused inductive bias rather than to manual mask annotation, unless explicitly stated otherwise. The lesion-supervised student is a separate comparison baseline in in Section 4.5 that is trained with ground-truth masks and is used only for the localization analysis.

To achieve region-specific modulation of the channel attention by the lesion prior, a relative gain fusion mechanism is designed:

(3) $$\begin{array}{c} A=\mathrm{clamp}(A_{data}⊙(1.0+g\cdot ({\overset{\sim }{A}}_{lesion}-0.5)),0,1)\; \end{array},$$

where ${\overset{\sim }{A}}_{lesion}$ is $A_{lesion}$ broadcast to match the channel dimension of $A_{data}$, and g is a learnable scalar controlling the strength of lesion guidance. Unlike direct multiplication, which may completely suppress background activations, the relative gain mechanism introduces an additive shift scaled by g and centered at 0.5. This ensures that background regions retain at least 50% of their original attention weight, preventing gradient vanishing and preserving discriminative information from non-lesion areas.

The fused attention map is added to the original features via a residual connection: $X_{enhanced}=X⊙A+X$. Subsequently, a 1 × 1 convolution compresses the channel dimension to half, followed by element-wise filtering using a gating signal generated from the learned lesion-aware attention map $A_{lesion}$ through an independent 1 × 1 convolution:

(4) $$\begin{array}{c} X_{out}=Conv_{1\times 1}^{C\to C/2}(X_{enhanced})⊙\sigma (Conv_{1\times 1}(A_{lesion})) \end{array}.$$

LaFEC outputs three tensors: $X_{enhanced}$ for downstream convolutional stages, $X_{out}$ as the distillation carrier for intermediate feature alignment, and the fused attention map, which is passed to LaLT and the loss functions. During distillation, the learned lesion-aware attention map A directly participates in the spatially weighted feature distillation loss, with gradients flowing normally back to the lesion localization branch (no detach operation is applied).

Although the teacher model embeds LaFEC after both Stage 2 and Stage 3, the student model employs LaFEC only after Stage 3, with Stage 2 replaced by a lightweight 1 × 1 projection layer. This asymmetric design is empirically motivated: ablation experiments in Section 4.4 demonstrate that deploying full LaFEC at Stage 2 in the lightweight student degrades accuracy by 0.48 percentage points (pp), from 91.90% to 91.42%. The rationale is twofold: (i) Stage 2 features lack sufficient semantic abstraction for reliable learned lesion-aware attention map generation, rendering the lesion-guided spatial modulation error-prone; and (ii) the student’s limited capacity cannot accommodate the parameter overhead and gradient competition of dual-branch LaFEC at shallow layers without incurring an information bottleneck. The 1 × 1 projection layer performs channel alignment without spatial modulation, preserving the integrity of low-level texture and edge information for downstream LaFEC and Transformer processing. This observation aligns with the hierarchical nature of CNN representations: lesion priors are more effective when applied to semantically mature features (Stage 3) rather than shallow features (Stage 2), as confirmed by Section 4.4.

### 3.2. LaLT: Lesion-Aware Lightweight Transformer Head

LaLT incorporates lesion awareness through two core designs: lesion-guided smooth bias attention and lesion token interaction. LaLT receives the compressed feature map from the student’s Stage 3 after LaFEC processing, converts it into non-overlapping patch tokens via a 2 × 2 convolution with stride 2, and then concatenates three learnable special tokens: a classification token (CLS), a distillation token, and a lesion token (newly introduced) that aggregates high-level lesion semantics and is aligned with the teacher’s counterpart during distillation.

Lesion-guided smooth bias attention: The learned lesion-aware attention map $A_{lesion}$ from LaFEC is downsampled to the patch level, yielding $M_{lesion}$. A smooth modulation coefficient is then computed via a sigmoid function with temperature parameter τ. We denote the temperature scaling parameter as τ (τ = 4 in all experiments unless otherwise specified), used for both the smooth modulation coefficient in Equation (5) and the softened output distributions in Equation (7):

(5) $$\begin{array}{c} s=\sigma \left(\left(M_{lesion}-0.5\right)\cdot \tau \right) \end{array}.$$

Within the window self-attention computation, the smooth modulation coefficient s is multiplied by a learnable bias strength ω and added as a bias term to the original attention scores, thereby dynamically weighting lesion regions. Specifically, for query token i and key token j, the attention weight is computed as:

(6) $$\begin{array}{c} \mathrm{Attention}\left(i,j\right)=Softmax\left(\frac{q_{i}\cdot k_{j}^{T}}{\sqrt{d}}+\omega \cdot b_{j}\right) \end{array},$$

where $q_{i}$ and $k_{j}^{T}$ are the query and key vectors of tokens i and j, respectively, *d* is the dimension of each attention head, and $b_{j}$ is the smooth modulation coefficient associated with key token j, obtained by applying Equation (5) to the downsampled lesion attention map.

### 3.3. LaHMD: Lesion-Aware Hierarchical Multi-Stage Distillation

LaHMD imposes synergistic constraints on the student model at three levels: output, feature, and semantic.

Output-level distillation: Let the teacher and student logits be $z^{T}$ and $z^{S}$, respectively, and let τ be the temperature parameter. The output-level distillation loss is measured by the Kullback–Leibler divergence:

(7) $$\begin{array}{c} L_{logit}=\mathrm{KL}(\sigma (z^{T}/\tau )∥\sigma (z^{S}/\tau )) \end{array}.$$

Spatially weighted hierarchical feature distillation: For each shared convolutional distillation stage j (Stage 2 and Stage 3), the compressed feature representations $F_{t}^{j}$ and $F_{s}^{j}$ are extracted, along with their respective lesion attention masks $M_{t}^{j}$ and $M_{s}^{j}$ derived from the LaFEC module (downsampled to match the feature map dimensions). The spatially weighted feature distillation loss applies each model’s own lesion attention mask to its respective feature map before computing the alignment residual. For feature distillation, each stage-wise loss is normalized by the feature map size:

(8) $$L_{feat}^{\left(j\right)}=\frac{1}{C_{j}H_{j}W_{j}}{\left\|M_{t}^{j}⊙F_{t}^{j}-M_{s}^{j}⊙F_{s}^{j}\right\|}_{2}^{2},$$

where $F_{t}^{j}$ and $F_{s}^{j}$ denote the aligned features after necessary projections, $M_{t}^{j}$ and $M_{s}^{j}$ denote the modulation masks used for feature alignment, and $C_{j}$, $H_{j}$, $W_{j}$ are the channel number, height, and width at stage j.

For Stage 2, the student does not apply LaFEC and therefore does not produce its own lesion attention mask. Accordingly, the student-side Stage-2 mask is defined as an identity map, $M_{s}^{2}=1$. In other words, only the teacher-side lesion attention map $M_{t}^{2}$ modulates the teacher feature at this stage, while the student feature remains unweighted. This design keeps the Stage-2 alignment well-defined without introducing an additional student-side lesion predictor. It is also consistent with our motivation that Stage-2 features are relatively low-level and may not provide sufficient semantic abstraction for learning a reliable lesion prior. At Stage 3, each model uses its own learned lesion attention mask.

The stage-wise feature distillation losses are then aggregated as:

(9) $$\begin{array}{c} L_{feat}=\sum_{j=2}^{3}\lambda_{j}\cdot L_{feat}^{\left(j\right)},{\;\;\lambda }_{j}=10 \end{array},$$

where $\;\lambda_{j}=min(\frac{\lambda_{base}}{\gamma_{j}},\lambda_{max})$, $\gamma_{j}=S_{j}/T_{j}$ is the ratio between the student’s and the teacher’s compressed channel counts at stage j, and $\lambda_{base}$ and $\lambda_{max}$ are set to 10 and 20, respectively. With the channel configuration used in this paper, $\gamma_{j}=1$ for both stages and hence $\lambda_{j}=10$. Because teacher and student features are projected to matched channel dimensions before Equation (8), and each loss term is normalized by $C_{j}H_{j}W_{j}$, a uniform weight provides stable training without introducing additional stage-dependent hyperparameters.

Construction of the teacher lesion token. Since the teacher is a pure CNN and does not contain an explicit Transformer token structure, its lesion-level semantic representation is constructed from the Stage-3 LaFEC output rather than produced by a Transformer head. Let $F_{t}^{3}$ in $R^{C_{t}\times H\times W}$ denote the teacher’s Stage-3 LaFEC feature map and $M_{t}^{3}$ in $[0,1{]}^{C_{t}\times H\times W}$ denote the channel-wise learned lesion attention map produced by LaFEC. The teacher lesion representation is obtained by lesion-modulated global average pooling:

(10) $$z_{t}^{lesion}=GAP(F_{t}^{3}⊙M_{t}^{3})\in R^{C_{t}}.$$

It is then mapped to the student lesion-token dimension by a linear projection:

(11) $$T_{t}^{lesion}=W_{t}z_{t}^{lesion}+b_{t}\in R^{d_{s}},{\;\;d}_{s}=192.$$

In our implementation, both the teacher-side representation and the student lesion token have dimension $d_{s}$ = 192. The projection layer is independent of the teacher’s classification head and is used only to provide the teacher-side target for lesion-token-level distillation. The teacher does not use a LaLT head. Implementation details of the projection heads, token initialization, and training protocol are provided in Appendix A (Appendix A.3).

Lesion token alignment. The lesion-token-level distillation loss is implemented as the average of an element-wise MSE term and a cosine-distance term:

(12) $$L_{token}=\frac{1}{2d_{s}}{\left\|T_{s}^{lesion}-T_{t}^{lesion}\right\|}_{2}^{2}+\frac{1}{2}(1-cos(T_{s}^{lesion},T_{t}^{lesion})).$$

The first term is the mean squared error between the $d_{s}$-dimensional token vectors, while the second term encourages directional semantic consistency.

The student lesion token is a learnable vector $T_{s}^{lesion}$ in $R^{d_{s}}$. It is initialized from a standard normal distribution and optimized jointly with the student network. Guided by the lesion-aware map produced by LaFEC, this token is updated through the LaLT head to capture lesion-level semantic information for classification. Since the teacher-side lesion representation is projected to the same dimension, Equation (12) directly aligns the student and teacher lesion tokens without requiring an additional student-side projection.

Total loss: The student model is trained with the following joint optimization objective:

(13) $$\begin{array}{c} L_{total}=(1-\alpha )L_{CE}+\alpha L_{logit}+\beta L_{feat}+\gamma L_{lesion} \end{array},$$

where $L_{total}$ denotes the total training loss; α, β, γ are scalar weighting coefficients that balance the contribution of each loss term; $L_{CE}$ represents the cross-entropy classification loss; $L_{feat}$ denotes the spatially weighted feature distillation loss between teacher and student intermediate representations; $L_{logit}$ is the output-level Kullback–Leibler divergence loss; and $L_{lesion}$ is the lesion token consistency loss computed over lesion-relevant attention tokens. From the perspective of imaging science, LaFEC directly contributes to image processing (feature enhancement and compression), LaLT contributes to image understanding (interpretable attention modeling), and LaHMD encourages the optimization process to prioritize lesion regions over purely global feature alignment.

In Equation (13), α controls the trade-off between the hard-label cross-entropy loss and the output-level logit distillation loss, while β and γ weight the feature-level and lesion-token-level distillation terms, respectively. In our experiments, we set α = 0.5, β = 4.0, and γ = 0.3. Although β is numerically larger than the other coefficients, the feature distillation term is first normalized by the stage weights ${\;\lambda }_{j}$ in Equation (9), which prevents any single feature stage from dominating the total loss. Empirically, the normalized feature loss and the logit distillation loss remain on comparable numerical scales during training. We also observed stable performance when varying β around 4.0, indicating that the final result is not sensitive to a narrow weight choice.

## 4. Experiments and Results

### 4.1. Datasets and Preprocessing

HAM10000 [43] contains 10,015 dermoscopic images (450 × 600 pixels) of seven skin lesion classes: dermatofibroma (DF), actinic keratoses and intraepithelial carcinoma (AKIEC), benign keratosis (BKL), melanoma (MEL), vascular lesions (VASC), melanocytic nevi (NV), and basal cell carcinoma (BCC). The dataset is highly imbalanced: NV has 6705 images (over 60%), while DF has only 115 images. Following the split of Datta et al. [44], 9187 samples are used for training and 828 for testing. While this allows fair comparison with prior works, we acknowledge that it does not enforce lesion-level separation, which may yield optimistic accuracy estimates due to potential lesion-level leakage. To quantify this bias, we additionally evaluate LaHKD under a lesion-disjoint protocol (see Appendix A.2).

Brain Tumor MRI [45] integrates Figshare, SARTAJ, and Br35H, totaling 7023 brain MRI images covering four classes: no tumor, pituitary tumor, meningioma, and glioma. Each class has more than 1600 samples, with a relatively balanced distribution. The training set contains 5712 samples and the test set contains 1311 samples. Because this benchmark aggregates data from multiple public sources, scanner variability and acquisition protocols are not explicitly standardized across the dataset.

All images were resized to 224 × 224 and normalized using the ImageNet mean and standard deviation ([0.485, 0.456, 0.406] and [0.229, 0.224, 0.225]). No 3D volumetric processing, skull stripping, or manual label cleaning across the integrated sources was performed.

The brain tumor MRI classification benchmark does not provide pixel-level lesion annotations. To enable exploratory localization analysis, we recovered ground-truth lesion masks by matching test images from the classification benchmark to images in the SciDB segmentation dataset [46], which provides pixel-level masks for the Figshare subset. Because the classification benchmark includes images originating from the same Figshare source, we performed cross-dataset image matching to identify corresponding images and transfer the available masks. Specifically, all candidate images were resized to a standardized resolution and converted to grayscale before perceptual hashes were computed. Candidate matches were identified based on hash distance, while horizontal flips were also considered to account for mirrored duplicates introduced during dataset construction. When a match was found, the corresponding SciDB mask was geometrically transformed as needed to ensure pixel-level alignment with the classification image. Unmatched or ambiguous cases were excluded from localization evaluation. This procedure yielded 180 valid test images with aligned lesion masks.

**Figure A1:**
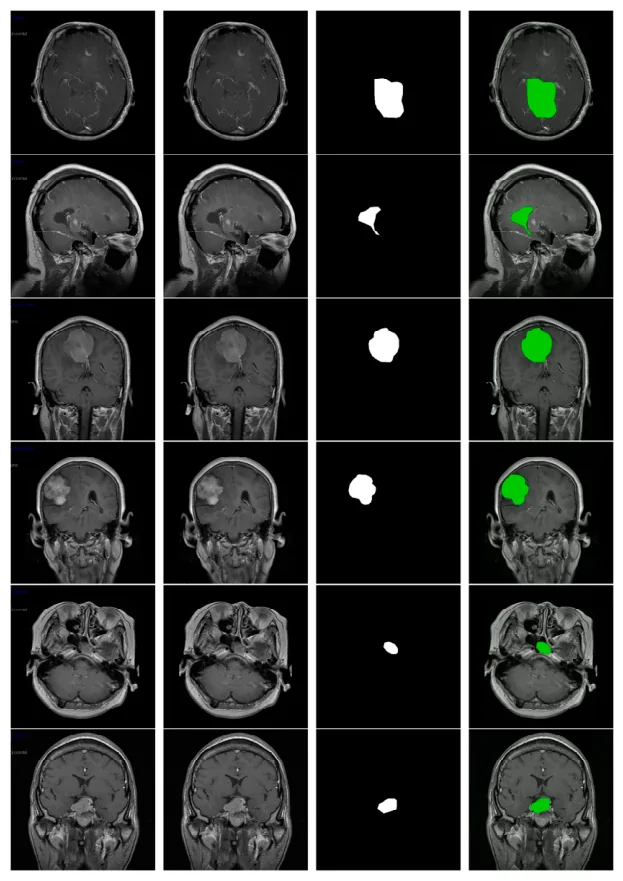
Representative matched MRI image pairs between the classification benchmark and the SciDB segmentation dataset. From left to right: (1) the original MRI slice from the classification benchmark; (2) the corresponding matched slice from the SciDB segmentation dataset; (3) the binary lesion mask, in which the white region denotes the tumor area; and (4) the overlay of the lesion mask (green) onto the original MRI slice to verify spatial alignment.

To improve transparency, we report the total number of test images, the number of successfully matched images, the number of excluded images, and the final sample size used for localization analysis in Appendix A.1. Because these lesion masks were recovered through cross-dataset image matching rather than provided directly within the classification benchmark, the MRI localization results should be interpreted as exploratory and with caution. A subset of matched image pairs was further checked manually to verify visual consistency between the classification images and the recovered segmentation masks. The manual verification results are reported in Appendix A.1, and representative matched examples are shown in Figure A1.

Because the Brain Tumor MRI benchmark may contain adjacent slices or near-duplicate images, we performed an additional near-duplicate analysis using dHash (Pillow ≥ 9.1.0). Images with Hamming distance ≤2 across training and test sets were regarded as near duplicates. Among 1311 test images, 573 images were identified as near duplicates and removed (see Appendix A.2), resulting in a clean test subset of 738 images. On this subset, LaHKD still achieved 99.62% accuracy, suggesting that the near-ceiling performance is not solely caused by train–test near-duplicate overlap. Nevertheless, because patient identifiers are unavailable, we acknowledge that strict subject-level separation cannot be guaranteed.

### 4.2. Implementation Details

LaHKD was implemented in PyTorch 2.3.1 (with CUDA 12.1) and trained on an NVIDIA GeForce RTX 4090 GPU (NVIDIA Corporation, Santa Clara, CA, USA). Unless otherwise specified, all models were trained from scratch without external pretrained weights, using a batch size of 32. The Teacher, Student (w/o KD), HDKD, and LaHKD were repeated with five random seeds: [42, 56, 123, 246, 2024], and other baselines were run once.

To improve generalization, we applied random flipping, rotation, color jittering, MixUp (α = 0.2), CutMix (α = 0.3), and label smoothing (0.1). This weak-augmentation configuration balances the regularization effect with lesion feature integrity, a practice widely validated in skin lesion classification tasks [47,48]. The teacher model was trained for 200 epochs using Adam with a fixed learning rate of 1 × 10^−3^ and no learning-rate schedule. For HAM10000, a training-only SMOTE procedure was used during teacher pretraining to mitigate severe class imbalance; no synthetic samples were used for student training or evaluation.

The student and all distillation baselines were trained for up to 200 epochs using AdamW with an initial learning rate of 1 × 10^−4^, weight decay 0.1, a cosine learning-rate schedule, and 10 warm-up epochs. For hyperparameter selection on HAM10000, the 9187-image training set was further divided into a stratified training subset (8268) and validation subset (919). The final models were then re-trained on the full 9187 training images and evaluated on the 828-image held-out test set. During validation-based selection, the student and distillation models used the checkpoint with the best validation accuracy under student-only inference, with early stopping (patience = 50). The teacher did not use early stopping. LaFEC does not require lesion annotations during training. The HAM10000 segmentation masks were used only for the localization evaluation in Section 4.5, including the auxiliary lesion-supervised comparison row they were not used to train LaHKD.

SMOTE was applied only after the train/test split and only for teacher pretraining. No synthetic samples were used in validation or testing, and the student was trained and evaluated without synthetic test data. We acknowledge that applying SMOTE in flattened pixel space may generate images that do not fully preserve realistic lesion morphology. Therefore, we treat SMOTE as a class-balancing strategy for teacher pretraining rather than as a clinically meaningful image-generation method. In future work, class-balanced sampling, class-weighted loss, or feature-space augmentation may provide more realistic alternatives.

All models were trained from scratch without ImageNet pretraining. This setting provides a controlled comparison of model architecture and distillation strategy, but may disadvantage data-hungry Transformer baselines. We therefore explicitly consider the comparison with ViT-family models as conservative and acknowledge this as a limitation. The HDKD baseline reported in this paper was reproduced by the authors using the same training and evaluation protocol as LaHKD, rather than directly quoted from the original publication. This ensures that the paired comparison between LaHKD and HDKD reflects differences in model design rather than differences in data splits or training schedules.

### 4.3. Experimental Results

We compared LaHKD with representative models from three categories: CNNs (MobileNetV2, MobileNetV3, EfficientNetB0), ViTs (PVT-Tiny, CrossViT-Ti, CSKD-Tiny), and hybrids (MobileViT-S, PVTv2-B0, CoAtNet-0, HDKD). All models were trained from scratch without pre-trained weights. Results are shown in Table 1.

Overall Performance. To improve result robustness, the main experiments for the Teacher, Student (w/o KD), HDKD, and LaHKD were repeated over five independent runs with different random seeds, and the mean ± standard deviation is reported for these methods. Statistical significance was assessed using paired t-tests when comparing LaHKD against the strongest distilled baseline, HDKD. Unless otherwise stated, results for other compared methods are from single runs, following their original implementation or standard evaluation settings. On the HAM10000 dataset, a paired t-test confirms that LaHKD’s improvement over HDKD is statistically significant (*p* < 0.01). On the Brain Tumor benchmark, where performance is near-saturated, the gain over HDKD is marginal and not statistically significant (*p* > 0.05); nevertheless, LaHKD achieves the highest numerical accuracy (99.89%) with the fewest parameters. Per-class metrics (Table 2, Table 3, Table 4 and Table 5) are reported from the best-performing single run to ensure consistent inter-class comparability; however, minor per-class variations should be interpreted within the variance bounds established in Table 1.

Per-Class Analysis (HAM10000). Table 2 and Table 3 detail class-level performance. LaHKD achieves weighted precision, recall, and area under the receiver operating characteristic curve (AUC) values of 93.7%, 93.6%, and 96.7%, respectively, with macro-AUC of 96.9% matching the teacher. For clinically critical classes, BKL precision exceeds that of HDKD by 3.7 percentage points, while MEL recall is also higher than that of HDKD, substantially reducing false negatives for malignancy. However, per-class metrics for rare categories such as DF (*n* = 6 in the test partition) and MEL (*n* = 34) should be interpreted with caution: with only six DF test samples, a single misclassification changes recall by 16.7 percentage points. Therefore, while the reported 100% recall and AUC for certain minority classes are mathematically correct, they are not statistically robust estimates. These figures should not be used to draw conclusions about real-world performance on underrepresented classes, and validation on larger, class-balanced cohorts is required before clinically meaningful conclusions can be drawn.

Brain Tumor Dataset. Table 4 and Table 5 show near-ceiling performance across all compared methods. The >99.6% accuracy across models on this benchmark indicates performance saturation, leaving limited room for substantial absolute improvement and suggesting that classification may rely on coarse global patterns or shortcut cues rather than fine-grained lesion morphology. Under these saturated conditions, LaHKD still achieves 99.89% accuracy with a perfect macro-AUC, matching the teacher’s performance while requiring 90.3% and 32.4% fewer parameters than CoAtNet-0 and HDKD, respectively. Therefore, the primary value of including this dataset is not to claim large performance margins, but to demonstrate that LaHKD maintains parameter-efficient classification under a different imaging modality and data distribution.

Table 2, Table 3, Table 4 and Table 5 report per-class metrics from the best-performing run to ensure that all per-class precision, recall, and AUC values are computed from a single internally consistent confusion matrix. However, these single-run per-class results should be interpreted descriptively rather than as statistically established class-wise improvements. The statistically supported conclusions are based on the five-run aggregate results in Table 1. In particular, differences on rare classes such as DF and VASC should be interpreted cautiously due to the small number of test samples.

### 4.4. Ablation Study

Table 6 presents an ablation study over nine configurations (C1–C9).

Architectural components. Starting from the baseline student model without distillation (C1, 89.37%), adding LaFEC alone improves Top-1 accuracy to 90.08% (+0.71 pp), while adding LaLT alone raises performance to 90.56% (+1.19 pp). Combining both modules (C4) further increases accuracy to 90.89% (+1.52 pp over C1), indicating that feature refinement and lesion-aware attention provide complementary benefits, although their effects are not fully additive. This suggests partial overlap in the information captured by the two modules. We hypothesize that LaFEC enhances lesion-relevant local representations, whereas LaLT improves long-range contextual modeling over lesion-relevant regions.

Distillation objectives. Building on C4, logit distillation alone (C5) yields a modest gain to 91.01% (+0.12 pp), whereas feature distillation alone (C6) produces a larger improvement to 91.46% (+0.57 pp), indicating that intermediate feature supervision is more effective than output-level transfer in this setting. Combining logit and feature distillation (C7) further increases performance to 91.52%, confirming that the two forms of knowledge transfer are complementary. Finally, adding lesion token supervision (C8, LaHKD) achieves the best result of 91.90%, demonstrating that semantic-level alignment between teacher and student lesion representations provides additional discriminative benefit beyond conventional logit- and feature-level distillation.

To disentangle architectural and distillation contributions, we compare three settings on the HAM10000 validation set. Under the identical LaFEC + LaLT backbone, conventional logit distillation (C5 in Table 6) achieves 91.01%, while our full LaHMD (C8) achieves 91.90%, yielding a +0.89 pp gain attributable to the hierarchical distillation objectives. Furthermore, applying LaHMD to the original student without LaFEC/LaLT (C9) reaches 91.17%, confirming that the architectural modules and the distillation strategy provide complementary gains. In this configuration, the Stage-3 feature term is inactive because the student produces no LaFEC features, and the lesion-token term uses a fallback token derived from the Distillation-based Feature Learning Transformer (DFLT) head of HDKD [16]; the remaining distillation terms still provide a moderate gain over the no-distillation baseline.

To verify the optimal deployment position of lesion-aware modules in the lightweight student, we compare two configurations: (i) LaFEC embedded after both Stage 2 and Stage 3, identical to the teacher architecture; and (ii) LaFEC only after Stage 3, with Stage 2 using a lightweight 1 × 1 projection for channel alignment. As shown in Table 7, the latter configuration achieves superior performance across all metrics.

The accuracy drop of 0.48 percentage points when deploying LaFEC at Stage 2 indicates that lesion-aware spatial gating at shallow layers is detrimental, rather than beneficial, to the lightweight student. This is due to two factors. First, Stage 2 features possess limited receptive fields and semantic abstraction; the learned lesion-aware attention map generated by LaFEC at this stage suffers from boundary ambiguity and false positives, causing erroneous suppression of discriminative features via the relative gain fusion (Equation (3)). Second, the student’s constrained capacity cannot accommodate the additional parameters and gradient competition introduced by a dual-branch LaFEC at Stage 2 without compromising core classification feature learning. The 1 × 1 projection preserves an “identity shortcut” for low-level information, allowing lesion-aware enhancement to be concentrated at Stage 3 where feature semantics are sufficiently abstracted for reliable mask generation. This asymmetric stage design is therefore a deliberate optimization under student capacity constraints, not a simplified omission.

### 4.5. Lesion-Focused Localization Analysis

To address the concern that Grad-CAM and learned lesion-aware maps are not directly comparable, we further conducted a matched localization analysis on the 828 HAM10000 test images with ground-truth masks. Both Grad-CAM and learned spatial priors were evaluated for the teacher and LaHKD student.

Evaluation metrics. To quantify spatial alignment, each spatial output (Grad-CAM or learned spatial prior) is resized to the input resolution and min–max normalized to [0, 1] per image. The Pointing Game measures the proportion of images in which the maximally activated pixel falls inside the ground-truth lesion mask. IoU@0.5 and Dice@0.5 are computed by binarizing the normalized map with a fixed threshold of 0.5 and comparing the resulting binary region with the ground-truth mask. Precision@Top-10% is the fraction of the top 10% highest-activated pixels that fall inside the ground-truth lesion. The Ratio (Inside/Outside) is the mean activation inside the lesion divided by the mean activation outside the lesion; larger values indicate more lesion-focused responses. The same normalization and thresholding procedures are applied to all compared spatial outputs.

The results show that the interpretation depends strongly on the type of spatial output. Teacher Grad-CAM achieves the highest Pointing Game score, indicating that the teacher often activates at least one point inside the lesion region; however, its IoU and Dice remain lower than those of the LaHKD learned spatial prior. In contrast, LaHKD Grad-CAM performs poorly, suggesting that post hoc gradients do not faithfully reveal the lesion-aware mechanism learned by LaHKD. We therefore no longer interpret Table 8 as a methodologically uniform Grad-CAM comparison, but as a comparison of model-specific spatial outputs. The attention map should not be interpreted as a full segmentation mask; it is a classification-oriented lesion-aware spatial prior optimized to improve recognition and distillation, rather than a pixel-level lesion segmentation output. This distinction is important because learned masks and Grad-CAM maps are fundamentally different forms of spatial explanation.

**Figure 3:**
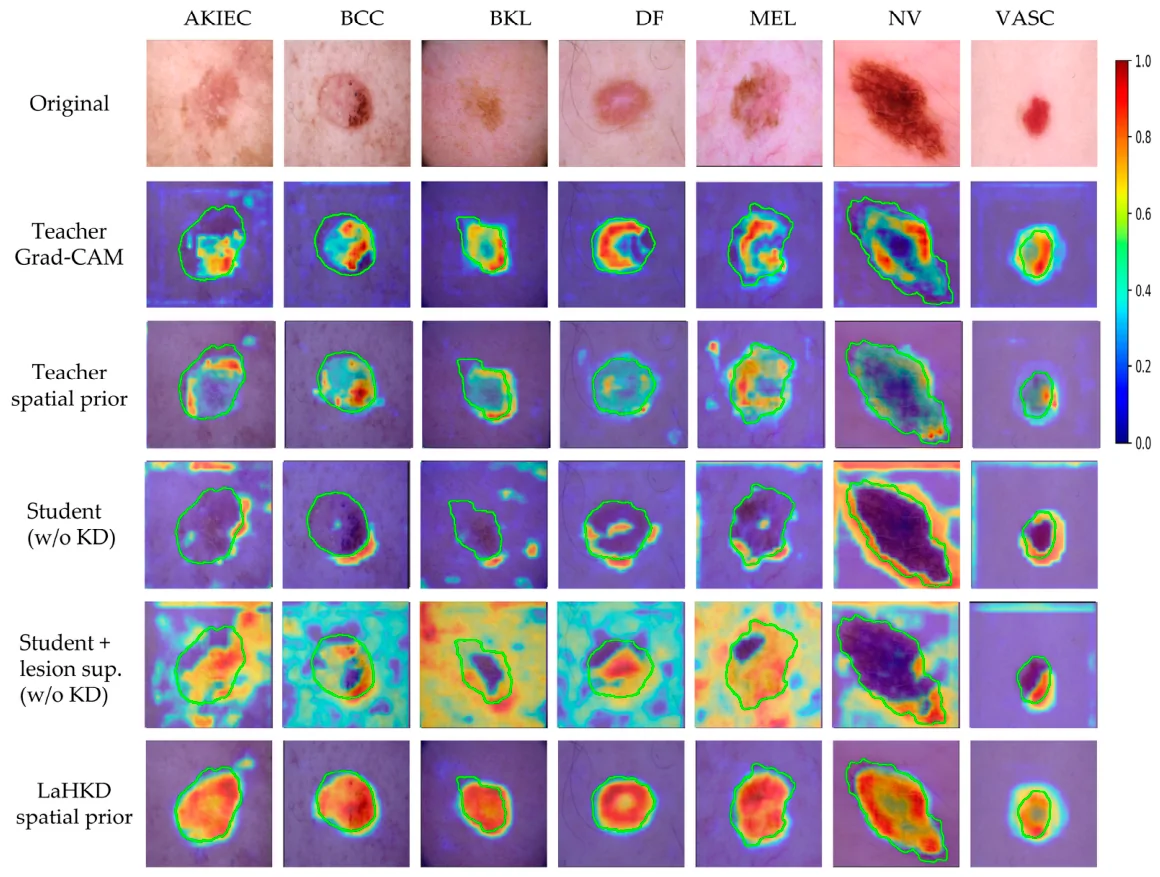
Qualitative comparison of spatial priors and Grad-CAM on HAM10000. The green contour denotes the ground-truth lesion boundary. The rightmost color bar indicates the relative activation intensity (0–1) using the jet colormap. Since each heatmap is min–max normalized independently for visualization purposes, the color intensity should be evaluated within each individual image to assess the spatial distribution, rather than directly comparing absolute values across different images.

Qualitative analysis on HAM10000 (Figure 3). The classification-only student exhibits diffuse activations across both lesion and background regions, suggesting weak lesion selectivity under image-level supervision alone. After adding lesion supervision without KD, the student becomes noticeably more lesion-focused, with reduced background activation and improved spatial concentration over the lesion area. However, its responses are still relatively coarse and less consistent along lesion boundaries. The teacher highlights lesion-related regions more reliably than the classification-only student, but still retains residual responses to hair structures, image boundaries, and surrounding skin patterns. By contrast, LaHKD produces the most spatially confined and contour-consistent attention maps, with stronger suppression of irrelevant background responses. These visual results are consistent with the quantitative findings in Table 8 and suggest that lesion-aware supervision and hierarchical distillation jointly improve lesion-focused representation learning in challenging dermoscopic cases, such as hair occlusion, low contrast, and irregular lesion borders.

For the brain tumor MRI benchmark, localization analysis was restricted to an auxiliary recovered-mask subset of 180 test images obtained through cross-dataset matching to the SciDB segmentation dataset. Unlike HAM10000, this benchmark does not provide native pixel-level lesion annotations; therefore, the MRI localization setting is necessarily indirect and weakly controlled. The resulting localization scores were generally low and did not show a consistent benefit from lesion-aware distillation (Table 9). Supplementary verification further indicated substantial noise in the recovered-mask subset: 47.8% of matched cases showed inconsistency between the expected tumor type encoded in the classification-image identifier and the segmentation-folder label of the recovered mask, and manual inspection found that only 6.7% were clearly visually consistent, whereas 47.8% were considered unreliable under stricter review. These observations indicate that the recovered MRI masks are markedly less reliable than native lesion annotations and therefore do not support strong localization claims.

At the same time, the MRI benchmark appears to be close to performance saturation for current deep models, where decision making may rely sufficiently on coarse global structure or shortcut cues rather than fine-grained lesion morphology. Under such conditions, explicit lesion-alignment constraints may still improve compact-model classification without necessarily yielding stable localization gains. In our experiments, LaHKD did not improve most localization metrics on the recovered-mask subset. We do not interpret this as evidence against the distillation mechanism itself; rather, it reflects both the limited reliability of the recovered-mask evaluation and the saturated nature of the task.

Although MobileNet-style models achieve higher raw throughput because of their highly optimized convolutional design (Table 10), their classification accuracy is notably lower than LaHKD on HAM10000. Compared with HDKD, LaHKD achieves both higher accuracy and higher throughput under the same hardware setting. Compared with the teacher, LaHKD reduces inference latency from 5.15 ms/image to 3.32 ms/image at batch size 1 and improves throughput from 571.2 images/s to 1960.8 images/s at batch size 32. These results show that the parameter and FLOP reductions achieved by LaHKD translate into practical inference efficiency. We note that the current latency evaluation is conducted on an RTX 4090 GPU. Evaluation on Jetson-class edge hardware is left for future work.

**Figure A2:**
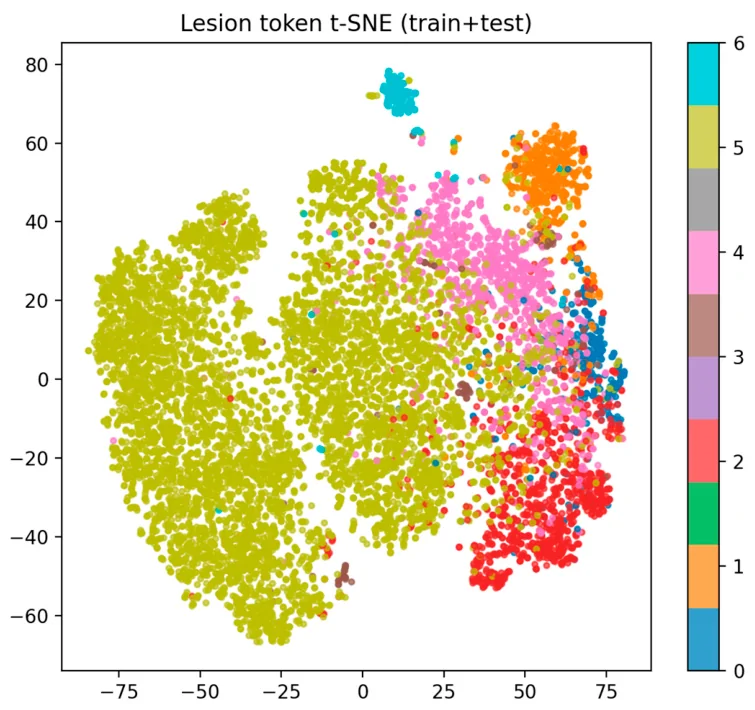
t-SNE visualization of lesion-token embeddings.

To further investigate the semantic information encoded in the lesion token, we conducted a linear probe experiment and t-distributed stochastic neighbor embedding (t-SNE) visualization. As detailed in Appendix A (Figure A2), the linear classifier trained on frozen lesion-token embeddings achieved 89.61% test accuracy, confirming that the lesion token captures class-discriminative semantics rather than acting merely as a spatial weighting vector.

## 5. Discussion

### 5.1. Why Lesion-Aware Hierarchical Distillation Improves Efficient Classification

The results across both datasets indicate that lesion-aware hierarchical distillation can improve the accuracy–efficiency trade-off of lightweight medical image classifiers. In the proposed framework, this gain is not attributable to a single component, but rather to the complementary interaction among lesion-aware feature enhancement, lightweight Transformer-based global modeling, and multi-stage knowledge transfer. Specifically, LaFEC strengthens local lesion-relevant representation learning while compressing features into a compact token set, LaLT promotes efficient long-range interaction among salient regions, and LaHMD transfers supervision from the teacher to the student at multiple semantic levels. Together, these modules help the student preserve diagnostically useful information under a strict parameter and computational budget.

A key advantage of this design is that it addresses a common weakness of lightweight student models in medical imaging, namely their tendency to overfit global appearance statistics or irrelevant background structures when trained only with image-level labels. By introducing lesion priors during training, LaHKD encourages the student to allocate representational capacity to spatially relevant regions rather than to shortcut cues alone. This is particularly valuable in heterogeneous distillation settings, where a compact CNN-Transformer student must absorb informative inductive biases from a stronger CNN teacher while maintaining high inference efficiency.

Importantly, the classification gains are more consistent than the localization gains across datasets. This suggests that lesion-aware distillation provides a generally useful regularization effect for compact medical image classification, even when task-dependent differences limit the reproducibility of spatial alignment improvements. From a practical perspective, these findings support LaHKD as an effective approach for building lightweight diagnostic models that remain competitive with much larger teachers.

### 5.2. Lesion-Focused Spatial Alignment in Dermoscopic Imaging

The HAM10000 experiments show that lesion-aware hierarchical distillation can substantially improve lesion-focused spatial alignment in dermoscopic imaging. Compared with the classification-only student, LaHKD produces much stronger overlap-based localization and more concentrated responses within lesion regions. The protocol-matched comparison in Table 8 further clarifies the source of this improvement. Specifically, the separately trained lesion-supervised comparison model already shows clear gains in Pointing Game, IoU, Dice, and precision, confirming that spatial supervision is a major determinant of localization quality in lightweight dermoscopic classifiers. However, the lesion-supervised student still remains far below the full LaHKD framework across all localization metrics. This indicates that the spatial advantage of LaHKD cannot be explained by annotation access alone; hierarchical teacher guidance contributes additional value by helping the student organize lesion-relevant features more effectively than mask supervision alone.

This result is particularly plausible in dermoscopy, where diagnosis depends heavily on lesion morphology, including shape, border irregularity, pigmentation structure, and local textural variation. In such settings, compact classifiers trained only with image-level labels are especially vulnerable to background confounders such as hair, ruler marks, illumination artifacts, and surrounding skin texture. The lesion-aware objectives in LaHKD appear to reduce this background interference and encourage the student to allocate its limited representational capacity to clinically meaningful regions. As a result, the student not only remains efficient, but also becomes more aligned with lesion-centered diagnostic cues.

At the same time, the comparison between LaHKD and the teacher must be interpreted carefully. In LaHKD, lesion awareness is learned end-to-end from classification supervision through the proposed lesion-aware objectives, whereas the teacher’s localization maps are derived post hoc from a model trained only for classification. The lesion-supervised student row in Table 8 is a separate baseline that uses ground-truth masks during training and is included only as a reference for the localization analysis. Therefore, the stronger localization of LaHKD on HAM10000 should not be interpreted as evidence of universal teacher–student localization superiority.

### 5.3. Boundary Conditions and Limitations of Spatial Evaluation

The MRI experiments reveal an important practical boundary condition of the proposed framework. Although LaHKD consistently improves parameter-efficient classification on the brain tumor MRI benchmark, it does not yield corresponding gains in localization metrics under the current evaluation setting. In contrast to the findings on HAM10000, this result suggests that the spatial benefit of lesion-aware distillation is not universal, but depends on the characteristics of the target task and the reliability of lesion supervision. When classification can be supported by coarse global structure, acquisition-related bias, or non-lesion contextual information, lesion-aware guidance may still regularize representation learning and improve classification performance without necessarily leading to measurable improvements in spatial alignment.

A major limitation of the MRI localization analysis is that the benchmark does not provide native pixel-level lesion annotations. To obtain a reference for spatial evaluation, we constructed an auxiliary subset by recovering lesion masks through cross-dataset image matching. However, this procedure introduced substantial uncertainty. Only a limited proportion of MRI samples could be matched, and supplementary manual verification indicated considerable semantic inconsistency and spatial noise in the recovered subset. Therefore, the resulting localization analysis should be interpreted as exploratory rather than confirmatory. Importantly, these recovered masks were used only for auxiliary evaluation and were not involved in model training, hyperparameter selection, or the primary performance claims of this study.

In addition, the Brain Tumor MRI benchmark exhibits near-ceiling classification performance across almost all compared models. This suggests that the task may be solvable using coarse global image patterns, scanner- or dataset-specific cues, or visually salient tumor-region differences, without requiring precise lesion-boundary localization. Moreover, because the dataset consists of 2D axial slices and does not consistently provide patient identifiers, subject-level or near-duplicate slice overlap cannot be fully excluded. Therefore, we interpret the Brain Tumor results conservatively.

In particular, LaHKD does not improve localization metrics on the recovered-mask MRI subset. This negative result does not necessarily contradict the lesion-aware design, because the recovered masks are not reliable ground-truth annotations and were not used for training. Instead, it indicates that classification accuracy on this benchmark should not be taken as evidence of fine-grained lesion localization. The main role of this dataset in our study is to evaluate whether LaHKD maintains compact and accurate classification under a different imaging modality, rather than to validate lesion-level interpretability.

Taken together, these findings indicate that the spatial benefit of lesion-aware hierarchical distillation is most clearly supported in the dermoscopic setting, where lesion annotations are directly available and spatial correspondence can be assessed more reliably. By contrast, the MRI experiments primarily support the parameter-efficient classification effectiveness of LaHKD across modalities, while also illustrating the practical limitations of localization analysis on a benchmark without native masks. Accordingly, the MRI localization results are reported only as exploratory evidence and are not used as a primary basis for the conclusions of this study.

### 5.4. Generalizability and Scope

The present results also help clarify the scope of lesion-aware supervision in medical image distillation. Across both datasets, LaHKD improves the performance of lightweight student models, indicating that lesion-aware hierarchical distillation can serve as a useful regularization strategy for compact medical image classification. However, the spatial effects are clearly task-dependent. On HAM10000, where lesion morphology is central to diagnosis and direct lesion supervision is available, LaHKD promotes more lesion-focused spatial responses and therefore supports more lesion-relevant representation learning. By contrast, comparable localization gains are not observed on the brain tumor MRI benchmark under recovered-mask evaluation.

We further examined the robustness of these conclusions under stricter evaluation settings. On HAM10000, LaHKD was additionally evaluated using a lesion-disjoint protocol, in which images from the same lesion were not shared across training and test sets. Under this stricter protocol, the accuracy decreased from 93.12% (seed 42) under the standard image-level split to 86.45%, indicating that image-level splitting provides an optimistic estimate of absolute generalization. Therefore, we retain the standard split for comparability with prior work, while reporting the lesion-disjoint split as a more conservative and annotation-consistent estimate.

On the cleaned Brain Tumor MRI subset (Appendix A.2), all models maintained near-saturated performance, suggesting that the high MRI accuracy was not solely caused by duplicate or near-duplicate samples. However, the performance gap between LaHKD and the compact student was small. We therefore interpret the MRI results mainly as evidence that LaHKD preserves high accuracy under a parameter-efficient architecture, rather than as evidence of statistically meaningful accuracy superiority on this saturated benchmark.

The near-saturated performance of the MRI benchmark further limits the headroom for demonstrating classification gains. Accordingly, the MRI experiments primarily support the parameter efficiency of LaHKD under a compact-model setting, rather than indicating broad remaining headroom on the benchmark itself.

Taken together, these findings suggest that lesion-aware distillation is most effective for spatial alignment when two conditions are satisfied: first, the diagnostic task genuinely depends on fine-grained lesion morphology; and second, reliable lesion annotations are available to guide and evaluate spatial behavior. When either condition is weak or absent, the benefit of lesion-aware supervision may primarily appear as improved classification efficiency rather than stronger localization evidence. Therefore, the current study does not claim universal teacher–student superiority in lesion localization across imaging modalities.

From a broader imaging perspective, LaHKD should be viewed as an efficient lesion-aware training framework whose benefits depend on task characteristics, rather than as a modality-independent solution for explainable localization. Future work should evaluate lesion-aware distillation on more rigorously curated benchmarks with native lesion masks, stronger control of acquisition bias, and tasks in which fine-grained lesion morphology is demonstrably required for prediction. Such settings will be important for determining when spatially informed distillation provides benefits beyond those of conventional compact classification training.

### 5.5. Limitations and Future Work

Several limitations of this study should be acknowledged. First, the standard HAM10000 split follows prior work at the image level; the lesion-disjoint evaluation in Appendix A.2 indicates that image-level splitting yields an optimistic estimate of absolute accuracy, so external validation on lesion-disjoint cohorts is needed. Second, the Brain Tumor MRI benchmark does not provide reliable patient identifiers, and strict subject-level separation cannot be guaranteed; the near-duplicate analysis in Appendix A.2 mitigates but does not eliminate this risk. Third, SMOTE was applied in flattened pixel space for teacher pretraining only, and although no synthetic samples were used for student training or evaluation, a systematic comparison with class-weighted loss or balanced sampling remains future work. Fourth, the MRI localization evaluation relies on recovered masks with substantial noise and should be regarded as exploratory. Fifth, the comparison focuses on HDKD as a reproduced medical KD baseline; conventional KD is included under the same student architecture (C5 in Table 6), while additional medical-imaging-specific KD baselines (e.g., DSP-KD [30]) are left for future work. Finally, inference efficiency was measured on an RTX 4090 GPU, and deployment-oriented evaluation on Jetson-class edge devices is left for future work. Addressing these limitations will be important for establishing the clinical value of lesion-aware distillation.

In addition, the scope of the spatial findings should be emphasized: the strongest spatial evidence is obtained on HAM10000, where reliable lesion annotations are available for evaluation, and the localization-related conclusions should therefore not be generalized to all medical imaging tasks. Because the present study evaluates two representative benchmarks, broader validation on curated multi-center datasets with native lesion masks remains necessary before these findings can be translated to other clinical settings.

## 6. Conclusions

This paper presented LaHKD, a lesion-aware hierarchical knowledge distillation framework for parameter-efficient medical image classification. Its central novelty is to treat lesion awareness as an explicit knowledge carrier rather than a post hoc explanation: lesion priors are injected into feature enhancement, architectural attention, and a hierarchical multi-stage distillation objective, and lesion-token-level semantics are aligned between a pure CNN teacher and a compact CNN–Transformer student. To the best of our knowledge, LaHKD is among the first lesion-aware hierarchical KD frameworks for compact medical image classification. The framework can incorporate lesion priors when such supervision is available, while retaining classification-only inference and without requiring lesion annotations for LaHKD training or testing.

On HAM10000, LaHKD achieves 93.33% top-1 accuracy with only 2.52 M parameters, significantly outperforming the reproduced HDKD baseline under paired testing while matching the teacher with 38.5% fewer parameters. Probing experiments show that the learned lesion token encodes class-discriminative information (89.61% linear-probe accuracy), and the matched analysis indicates that LaHKD’s localization advantage stems from its explicit learned spatial prior rather than from post hoc Grad-CAM behavior.

From a clinical perspective, LaHKD addresses two practical requirements of computer-aided diagnosis: high accuracy under strict parameter budgets, and interpretable, lesion-relevant evidence. Because the compact student jointly optimizes classification with an internal spatial prior, it can provide lesion-focused explanations without relying on separate post hoc visualization tools. Measured efficiency gains—a 36% latency reduction at batch size 1 (5.15 → 3.32 ms/image) and a 3.4× throughput improvement at batch size 32 compared with the teacher—lower the hardware and energy barriers for deployment in resource-constrained clinical settings, particularly in dermoscopic screening, where lesion morphology is central to decision making.

The scope of these claims should be stated carefully. On the brain tumor MRI benchmark, LaHKD maintains near-ceiling classification accuracy with fewer parameters, but the task is close to saturation, patient-level separation cannot be fully guaranteed, and the recovered-mask localization evaluation provides only exploratory evidence. Spatial benefits are therefore most clearly supported when fine-grained lesion morphology matters and reliable lesion annotations are available. Future work will focus on patient-level validation on more rigorously curated benchmarks, class-balanced training strategies, additional conventional and medical-imaging-specific KD baselines, and edge-device deployment, with the goal of translating lesion-aware distillation into clinically validated computer-aided diagnosis systems.

## Figures and Tables

**Table 1:** Comparison with state-of-the-art methods on HAM10000 and Brain Tumor datasets.

	Model	Params	FLOPs	HAM10000 Top-1 Acc	Brain Tumor Top-1 Acc
ConvNets	MobileNetV2 [21]	2.2 M	0.32 G	90.70%	99.62%
	MobileNetV3-L [49]	4.2 M	0.23 G	90.46%	99.77%
	EfficientNetB0 [3]	4.0 M	0.41 G	90.70%	99.77%
	RegNetY-4.0 [50]	19.6 M	4.0 G	89.73%	99.62%
	Teacher model (ours) *	4.10 M	5.93 G	93.21 ± 0.29%	99.62 ± 0.08%
ViTs	PVT-Tiny [24]	12.7 M	1.88 G	87.80%	98.86%
	CrossViT-Ti [51]	6.7 M	1.6 G	87.44%	99.08%
	CSKD-Tiny [14]	5.5 M	1.1 G	89.86%	99.24%
Hybrid Models	MobileViT-S [8]	5.0 M	2.0 G	89.73%	99.62%
	PVTv2-B0 [25]	3.4 M	0.54 G	88.89%	99.08%
	CoAtNet-0 [7]	26 M	4.21 G	90.22%	99.85%
	Student (w/o KD) *	2.52 M	1.82 G	90.99 ± 0.40%	99.77 ± 0.06%
	HDKD [16] *	3.73 M	2.09 G	92.15 ± 0.31%	99.85 ± 0.05%
	LaHKD *	2.52 M	1.82 G	93.33 ± 0.42%	99.89 ± 0.04%

FLOPs: floating-point operations; Top-1 Acc: top-1 accuracy. * Results for Teacher, Student, HDKD, and LaHKD are reported as mean ± standard deviation over five independent runs. Other baseline models are reported from single runs under the same evaluation protocol. Two-sided paired t-tests were performed using identical seeds and data splits for Teacher, Student, HDKD, and LaHKD. On HAM10000, LaHKD significantly outperforms HDKD: mean difference = 1.18 percentage points, $t\left(4\right)=4.87$, p=0.008, 95% CI [0.51, 1.85] pp. LaHKD also significantly outperforms the non-distilled student: mean difference = 2.34 pp, $t\left(4\right)=12.31$, p=0.001, 95% CI [1.82, 2.87] pp. The difference between LaHKD and the teacher is not statistically significant: mean difference = 0.12 pp, $t\left(4\right)=0.43$, p=0.689, 95% CI [−0.66, 0.89] pp. On the Brain Tumor benchmark, all methods achieve near-ceiling performance; therefore, small numerical differences should not be interpreted as statistically or clinically meaningful without further patient-level validation.

**Table 2:** Precision, recall, and area under the receiver operating characteristic curve (AUC) on HAM10000 (teacher, student, LaHKD).

Class	Precision (%)			Recall (%)			AUC (%)			#
	Teacher	Student	LaHKD	Teacher	Student	LaHKD	Teacher	Student	LaHKD	
AKIEC	85.7	71.4	87.5	58.7	65.2	60.9	94.2	90.0	91.7	23
BCC	95.5	85.7	87.0	80.8	69.2	76.9	98.5	96.9	99.4	26
BKL	72.2	80.0	78.3	78.8	78.8	81.8	95.5	94.4	94.7	66
DF	100.0	75.0	75.0	100.0	100	100	100.0	100	100	6
MEL	72.7	54.8	68.4	70.6	67.6	76.5	93.6	91.1	96.0	34
NV	97.0	96.2	97.1	98.3	96.4	97.6	96.4	94.2	97.0	663
VASC	88.9	100	100	80.0	70.0	80.0	99.9	100	99.7	10
Avg.	87.4	80.4	84.8	81.0	78.2	82.0	96.9	95.2	96.9	828
W.Avg.	93.6	92.1	93.7	93.8	91.8	93.6	96.5	94.2	96.7	828

**Table 3:** Comparison of precision, recall, and AUC among CoAtNet-0, HDKD, and LaHKD on HAM10000.

Class	Precision (%)			Recall (%)			AUC (%)			#
	CoAtNet-0	HDKD	LaHKD	CoAtNet-0	HDKD	LaHKD	CoAtNet-0	HDKD	LaHKD	
AKIEC	100	68.8	87.5	39.1	47.8	60.9	96.6	96.5	91.7	23
BCC	62.1	63.2	87.0	69.2	93.3	76.9	98.7	98.6	99.4	26
BKL	75.0	74.6	78.3	68.2	75.8	81.8	94.6	96.3	94.7	66
DF	75.0	75.0	75.0	50.0	100	100	95.7	99.8	100	6
MEL	44.4	73.1	68.4	47.1	55.9	76.5	94.2	96.1	96.0	34
NV	95.0	97.4	97.1	97.7	97.1	97.6	96.4	98.4	97.0	663
VASC	100	75.0	100	80.0	90.0	80.0	100	99.9	99.7	10
Avg	78.8	75.3	84.8	64.5	80.0	82.0	96.6	98.0	96.9	828
W.Avg	90.4	92.3	93.7	90.2	92.1	93.6	96.3	98.1	96.7	828

**Table 4:** Precision, recall, and AUC on Brain Tumor MRI (teacher, student, LaHKD).

Class	Precision (%)			Recall (%)			AUC (%)			#
	Teacher	Student	LaHKD	Teacher	Student	LaHKD	Teacher	Student	LaHKD	
Glioma	100.0	100.0	100	99.3	99.7	99.7	100.0	99.8	100.0	300
Meningioma	99.0	99.0	99.4	99.3	100.0	100	100	99.9	100.0	306
No tumor	99.5	100.0	100	100.0	100.0	100	100.0	100.0	100.0	405
Pituitary	100.0	100.0	100	99.7	99.3	99.7	100.0	100.0	100.0	300
Avg	99.6	99.8	99.9	99.6	99.8	99.9	100.0	99.9	100.0	1311
W.Avg	99.6	99.8	99.9	99.6	99.8	99.9	100.0	99.9	100.0	1311

**Table 5:** Comparison of precision, recall, and AUC among CoAtNet-0, HDKD, and LaHKD on Brain Tumor MRI.

Class	Precision (%)			Recall (%)			AUC (%)			#
	CoAtNet-0	HDKD	LaHKD	CoAtNet-0	HDKD	LaHKD	CoAtNet-0	HDKD	LaHKD	
Glioma	100	100	100	99.7	100	99.7	100	100	100.0	300
Meningioma	99.4	99.7	99.4	100	99.7	100	100	99.9	100.0	306
No tumor	100	99.8	100	100	100	100	100	100	100.0	405
Pituitary	100	100	100	99.7	99.7	99.7	100	100	100.0	300
Avg	99.8	99.9	99.9	99.8	99.8	99.9	100	100	100.0	1311
W.Avg	99.8	99.8	99.9	99.8	99.8	99.9	100	100	100.0	1311

**Table 6:** Ablation study on HAM10000 validation set.

Config	LaFEC	LaLT	Distillation Strategy	Top-1 Acc (%)
C1 (Baseline)	✗	✗	None (CE only)	89.37
C2	✓	✗	None (CE only)	90.08
C3	✗	✓	None (CE only)	90.56
C4	✓	✓	None (CE only)	90.89
C5	✓	✓	Logit distillation only	91.01
C6	✓	✓	Feature distillation only	91.46
C7	✓	✓	Logit + Feature distillation	91.52
C8 (LaHKD)	✓	✓	Logit + Feature + Lesion Token	91.90
C9	✗	✗	LaHMD (logit + feature + lesion token)	91.17

✓ indicates the module is used, ✗ indicates it is not used.

**Table 7:** Effect of Stage 2 compression strategy in the student model (evaluated on the HAM10000 validation set).

Stage 2 Module	Stage 3 Module	Top-1 Acc (%)	Pointing Game	IoU@0.5	Dice@0.5
LaFEC	LaFEC	91.42	0.693 ± 0.462	0.514 ± 0.199	0.653 ± 0.198
1 × 1 Projection	LaFEC	91.90	0.739 ± 0.453	0.532 ± 0.188	0.672 ± 0.183

**Table 8:** Matched localization comparison on HAM10000 using different spatial outputs.

Spatial Output	Pointing Game	IoU@0.5	Dice@0.5	Precision@Top-10%	Ratio (Inside/Outside)
Teacher Grad-CAM	0.954 ± 0.209	0.365 ± 0.190	0.505 ± 0.209	0.858 ± 0.205	7.745 ± 3.774
Teacher learned spatial prior	0.620 ± 0.485	0.216 ± 0.196	0.317 ± 0.241	0.765 ± 0.256	18.312 ± 19.173
Student (w/o KD)	0.188 ± 0.391	0.089 ± 0.069	0.156 ± 0.109	0.196 ± 0.185	0.895 ± 0.474
Student + lesion supervision (w/o KD)	0.469 ± 0.499	0.215 ± 0.131	0.337 ± 0.162	0.420 ± 0.233	1.110 ± 0.488
LaHKD Grad-CAM	0.194 ± 0.396	0.059 ± 0.117	0.094 ± 0.164	0.166 ± 0.260	1.702 ± 26.155
LaHKD learned spatial prior	0.739 ± 0.453	0.532 ± 0.188	0.672 ± 0.183	0.808 ± 0.247	13.985 ± 9.755

**Table 9:** Exploratory localization analysis on the recovered-mask MRI subset.

Metric	Teacher (Grad-CAM)	Student (w/o KD)	LaHKD (Ours)
Pointing Game	0.011 ± 0.105	0.050 ± 0.218	0.028 ± 0.164
IoU@0.5	0.019 ± 0.047	0.020 ± 0.040	0.016 ± 0.035
Dice@0.5	0.034 ± 0.078	0.037 ± 0.069	0.030 ± 0.062
Precision@Top-10%	0.022 ± 0.048	0.021 ± 0.048	0.020 ± 0.042
Ratio (Inside/Outside)	1.109 ± 1.255	0.840 ± 0.415	1.120 ± 1.083

**Table 10:** Measured inference latency and throughput on RTX 4090 GPU.

Model	Params	FLOPs	Batch = 1 FP32 ms/img	Batch = 1 img/s	Batch = 32 FP32 ms/img	Batch = 32 img/s	Batch = 64 FP32 ms/img	Batch = 64 img/s
Teacher	4.10 M	5.93 G	5.15	194.2	1.75	571.2	1.86	538.4
Student w/o KD	2.52 M	1.82 G	3.28	298.9	0.51	1960.4	0.54	1851.1
HDKD	3.73 M	2.09 G	3.81	262.7	0.58	1714.5	0.62	1610.7
LaHKD	2.52 M	1.82 G	3.32	301.2	0.51	1960.8	0.54	1851.9
MobileNetV2	2.2 M	0.32 G	1.94	515.5	0.13	7810.0	0.15	6848.4
PVTv2-B0	3.4 M	0.54 G	2.22	451.2	0.22	4599.5	0.21	4708.6
CoAtNet-0	26 M	4.21 G	3.26	306.8	0.71	1415.4	0.73	1372.2

## Data Availability

The datasets used in this study are publicly available. The HAM10000 dataset can be accessed at https://dataverse.harvard.edu/dataset.xhtml?persistentId=doi:10.7910/DVN/DBW86T (accessed on 1 September 2026), and the Brain Tumor MRI dataset can be accessed at https://www.kaggle.com/datasets/masoudnickparvar/brain-tumor-mri-dataset (accessed on 1 September 2026). The source code of the proposed framework is available from the corresponding author upon reasonable request.

## Appendix A

### Appendix A.1. Cross-Dataset Matching and Verification

Because the MRI classification benchmark lacks native lesion masks, we constructed an auxiliary localization subset through cross-dataset image matching. Table A1 summarizes the subset construction process. Supplementary verification (Table A2 and Table A3) revealed substantial semantic and spatial noise in the recovered matches. Therefore, the corresponding MRI localization analysis is reported only as exploratory evidence and is not used as a primary basis for the study’s conclusions.

Accordingly, the MRI localization results reported in the main text should be interpreted only as exploratory evidence under a weakly controlled evaluation setting, rather than as a definitive localization benchmark. Representative recovered examples are shown in Figure A1.

**Table A1:** Summary of auxiliary MRI localization subset construction.

Item	Count
Total MRI test images	1311
Successfully matched images	180
Excluded images (unmatched or ambiguous)	1131
Final localization subset	180

**Table A2:** Identifier-level consistency check for recovered MRI image–mask pairs.

Expected Image Type → Actual Segmentation Folder	Count	Proportion of Matched Subset
Glioma → Meningioma	60	33.3%
Meningioma → Glioma	9	5.0%
Glioma → Pituitary	9	5.0%
Meningioma → Pituitary	7	3.9%
Pituitary → Glioma	1	0.6%
Total inconsistent cases	86	47.8%

**Table A3:** Manual visual verification of recovered MRI image–mask pairs.

Verification Outcome	Count	Proportion
Clearly visually consistent	12	6.7%
Minor differences but acceptable approximate alignment	82	45.6%
Unreliable under stricter visual inspection	86	47.8%
Total manually inspected matched pairs	180	100.0%

### Appendix A.2. Near-Duplicate Analysis and Lesion-Disjoint Evaluation

Table A4, Table A5, Table A6 and Table A7 summarize the near-duplicate analysis and the lesion-disjoint evaluation.

**Table A4:** Near-duplicate analysis on the Brain Tumor MRI benchmark.

Item	Count	Ratio
Total MRI test images	1311	100%
Cross train-test near duplicates	573	43.7%
Exact hash matches, distance = 0	437	33.3%
Hamming distance = 1	75	5.7%
Hamming distance = 2	61	4.7%
Clean test subset	738	56.3%

**Table A5:** Class-wise distribution of removed near-duplicate images.

Class	Original Test	Removed Near Duplicates	Clean Test
Glioma	300	28	272
Meningioma	306	123	183
No tumor	405	378	27
Pituitary	300	44	256
Total	1311	573	738

**Table A6:** Classification performance on the clean MRI test subset.

Model	Accuracy	Macro Recall	Macro F1	Macro AUC
Teacher	99.32%	99.45%	98.63%	99.98%
Student w/o KD	99.59%	99.72%	99.66%	99.88%
LaHKD	99.62%	99.72%	99.66%	99.91%

**Table A7:** Performance comparison between image-level and lesion-level splits on HAM10000 (seed 42).

Split Protocol	Granularity	Train/Test	LaHKD Top-1 Acc	Δ vs. Image-Level
Image-level [44]	Per-image	9187/828	93.12%	—
Lesion-level	Per-lesion	9018/997	86.45%	−6.67 pp

### Appendix A.3. Architectural Details and Lesion Token Analysis

Table A8 and Table A9 present the architectural details of the teacher and student models and the class-wise correlation between the learned lesion-aware attention maps and the ground-truth lesion masks, respectively.

**Table A8:** Channel dimensions and projection layers of teacher and student.

Model	num_Blocks	Channels	LaFEC Placement	Transformer Layers	Patch Size	Embedding Dimension	Heads × Dim	Lesion-Token Dim
Teacher	[6, 6, 9, 3]	[64, 96, 192, 256]	after Stages 2 and 3	N/A	N/A	N/A	N/A	192
Student	[2, 2, 3, 3]	[64, 96, 192, 192]	after Stage 3 only	2	(2, 2)	192	8 × 32	192

N/A: not applicable.

**Table A9:** Class-wise correlation between learned attention maps and ground-truth lesion masks on HAM10000.

Class	N	LaHKD Pearson	LaHKD IoU@0.5	Teacher Pearson	Teacher IoU@0.5
AKIEC	23	0.678	0.548	0.564	0.172
BCC	26	0.742	0.598	0.672	0.287
BKL	66	0.715	0.543	0.620	0.174
DF	6	0.806	0.656	0.764	0.263
MEL	34	0.735	0.546	0.664	0.320
NV	663	0.712	0.517	0.654	0.213
VASC	10	0.528	0.351	0.513	0.272

To examine what information is encoded in the lesion token, we froze the trained LaHKD model and trained a linear classifier on the extracted lesion-token embeddings. The linear probe achieved 89.61% test accuracy on HAM10000. This result is substantially higher than random chance and close to the full LaHKD classification accuracy, indicating that the lesion token contains class-discriminative semantic information rather than merely acting as a spatial weighting vector. In addition, the t-SNE visualization of lesion-token embeddings shows class-dependent clustering patterns, further suggesting that the lesion token captures lesion-relevant diagnostic semantics.
